# Investigating cow-calf productive performance under early and conventional weaning practices in south african beef cattle

**DOI:** 10.1016/j.vas.2025.100472

**Published:** 2025-06-27

**Authors:** Brent Damian Jammer, Willem Abraham Lombard, Henry Jordaan

**Affiliations:** Department of Agricultural Economics, University of the Free State, Bloemfontein 9300, South Africa

**Keywords:** Weaning management, Production efficiency, Decision-making, Weaning weight

## Abstract

Weaning age is a critical decision in beef cattle systems, ultimately influencing productivity and reproductive efficiency. This study investigated the productive performance of 152 Bonsmara cow-calf pairs under two distinct weaning practices: Early Weaning (EW) at 90 days and Conventional Weaning (CW) at 205 days. Data were obtained from the Arcadia Farmland cattle herd in the Vrede region of South Africa, comparing primiparous heifers with EW calves to a group of primiparous and second-time calving heifers with CW calves as a baseline comparison, reflecting limited research on EW in South Africa. EW calves received grower meal and natural grazing post-weaning, while CW calves depended on maternal milk and natural grazing. The farm's productive data indicated that CW achieved higher weaning weights per calf (+27 kg), highlighting productivity benefits. Conversely, EW reduced inter-calving periods (347 vs. 419 days), enhanced fertility, and improved herd reproductive efficiency, offering advantages in drought-prone settings. A General Linear Model (GLM) was applied to assess the influence of productive factors on 205-day calf weight. The model identified **weaning practice** and **dam calving weight** as significant predictors. Heavier dams produced heavier calves, likely due to better maternal conditions. These results highlight trade-offs between live weight gain (CW) and reproductive efficiency (EW), reinforcing the need for context-specific weaning strategies in South African beef systems.

## 1 Introduction

Beef cattle producers often face significant challenges in maintaining production efficiency in cattle due to climatic variability and economic pressures in farming operations, necessitating adaptations and innovation to ensure long-term sustainability (Spies, 2011). Limited pasture availability driven by dry climatic conditions further drives producers to make strategic decisions to alter their farm management strategies. This allows for optimizing natural resources to maintain cattle productivity continuously. Despite its critical role in calf growth and overall productivity, weaning management often needs producers' detailed attention and other aspects of farm management (Welk et al., 2024). According to Meagher et al. (2019), placing greater emphasis on weaning practices, particularly the timing of weaning, is essential for addressing resource optimization challenges for cattle productivity.

Weaning marks a significant milestone in a calf's life and often influences its growth and welfare (Eckert et al., 2015). Whalin et al. (2021) mentioned that the age at weaning has implications not only for calves but also for the long-term productivity of dams. Optimal management postpartum plays a key role, as producers need to ensure dams’ nutritional demands are maintained through adequate supplementation and grazing. Strategic decisions regarding weaning practices may enable producers to optimize grazing use by reducing dams' nutrient requirements, particularly under dry climatic conditions where pasture availability is restricted (Camargo et al., 2022). By carefully re-evaluating weaning age and practices, beef cattle producers can better address the interconnected challenges of climatic variability, pasture restrictions, and optimal productivity.

For example, early weaning (**EW**) is a practice that has been widely implemented in dairy, but particularly in beef cattle when pasture is of poor quality, the growing season is short, cows have a limited milk supply, and for first calving heifers (Meyers et al., 1999; Rust & Rutst, 2014 and Welk et al., 2024). Moreover, Savage (2011) stated that EW has the potential to be used for increased productivity, providing a range of land use options and decreasing on-farm production costs in beef cattle. Rust and Rust (2013) and Taylor et al. (2021) observed that EW improves calves' feed conversion ratios (FCR), leading to optimal growth rates and competitive weaning weights compared to calves that suckle for extended periods. In contrast, EW may lower calf weaning weights and increase operational costs within cow-calf production systems (Julien & Tess, 2002; Meagher et al., 2019). From the perspective of dams, Savage (2011) found that EW enhances dam performance through improved conception rates, as reduced lactation demands lower maintenance requirements. This improvement translates to greater overall productivity in production systems. Dams with strong maternal ability and optimal body condition scores (BCS) produce adequate milk that supports calf welfare, growth, and weight gain (Camargo et al., 2022). These findings highlighted that the improved performance of dams is a key factor for producers to consider when assessing the potential benefits of adopting EW practices.

In South Africa, calves are conventionally weaned (**CW**) between six and nine months, and in some cases, at most, twelve months (Strydom, 2008; Mare, 2018 and Van der Westhuizen et al., 2020). However, the concept of EW has primarily been adopted in the South African dairy sector (Muya & Nherera, 2014) and, to the author’s knowledge, has not yet been commonly practiced in beef cattle. Additionally, little research has reported EW (at 90 days) and its impact on managing beef cow-calf systems in South Africa. Although the EW of beef calves has been successfully explored outside South Africa, the climatic conditions differ significantly among countries, which may influence the productivity outcomes of beef cattle production systems (Vaz et al., 2022). These differences are often shaped by the unique characteristics of management practices and geographical regions, including cattle breeds well adapted to these conditions. Notably, in South Africa, where drought and variable pasture conditions are common, sufficient and cost-effective feed supply for EW calves up to 205 days poses a significant management consideration.

Moreover, producers in South Africa may not have extensively explored EW due to its limited adoption outside the dairy sector or minimal research evaluating its impacts on beef cow-calf systems with local breeds and climatic conditions. This significant research gap underscores the need to establish foundational insights into the productive performance of beef cows and calves under EW and CW systems in South Africa. Providing preliminary performance indicators can help producers make more informed decisions regarding the optimal weaning age for their calves. Camargo et al. (2022) highlighted that various factors influence the weaning age practices adopted by cow-calf producers, including income potential, weaning costs, herd maintenance, nutritional management, and, ultimately, the performance of both calves and dams. Hence, the primary objective of this study was to establish baseline data indications on the productive performance of Early Weaning (EW) practices in South African beef cattle production, a management strategy not yet widely implemented in the region.

This study addressed the knowledge gap on EW practices in South African beef cattle by providing preliminary insights into EW's potential benefits and challenges by comparing the productive performance of cow-calf pairs subjected to early weaning (90 days) and conventional weaning (205 days). The analysis included cows of varying reproductive stages, reflecting the practical realities of the investigated herd composition. Ultimately, the findings of this research aim to equip South African producers with evidence-based guidance on optimal weaning age decisions (EW or CW) by evaluating these practices and highlighting potential outcomes of EW weaning practices, particularly under local environmental conditions and management systems using an indigenous cattle breed.

## 2 Materials and methods

### 2.1 Data

#### 2.1.1 Cow and calf grouping

This study used production data from Arcadia Bonsmara Farmland, located in the Vrede region of the Free State Province, South Africa. The data represented one full year of records (one calving season) and included 152 Bonsmara cow-calf pairs. These pairs were grouped based on standard herd management practices rather than experimental treatments. Specifically, early-weaned (EW) calves were born to first-time heifers that had been bred early at 14–15 months, while conventionally weaned (CW) calves came from a mix of first- and second-parity cows.

*Grouping was therefore based on the farm's established management system:*

1. Early Weaning Group (W90): Calves weaned at 90 days, consisting of primiparous cows (first-time heifers calving between 24–35 months).
2. Conventional Weaning Group (W205): Calves weaned at 205 days, primarily consisting of a subset of first and mostly second-time calvers (calving between 28–38 months).

The comparison is thus observational and based on standard farm practice, making the study design retrospective and not a controlled randomized trial.

#### 2.1.2 Study context

The data was obtained from Arcadia Farmland, which, to the authors’ knowledge, is one of the first farms in South Africa to implement EW (90 days) in beef cattle exclusively for a group of first-time calving heifers. Arcadia Bonsmara applies early weaning to heifers bred at 14–15 months to support reproductive and developmental outcomes (Savage, 2011). While no formal randomization occurred, the farm's routine grouping strategy provides a natural division suitable for herd management. Thus, this study aimed to provide a performance-based baseline comparison of EW and CW outcomes under South African conditions, bearing in mind a key limitation of this study, which is the structural disparity between the groups.

#### 2.1.3 Herd management

The EW (90) group consisted of 76 dams and 76 calves (37 male and 39 female) that were weaned at 90 days with a minimum weaning weight of 86 kg. The CW group consisted of 76 dams and 76 calves (38 male and 38 female) that were weaned at 205 days with a minimum weight of 209 kg. **All cow-calf pairs grazed on natural pastures (Themeda triandra and Pennisetum clandestinum) and were stocked at a 2.5** **ha/ Large Livestock Unit (LSU) stocking rate on the Arcadia farmland in Vrede.** EW and CW cow-calf groups were kept in separate paddocks (two camps) on the same farm to avoid intermingling and maintain group identity. Following weaning, the **group** of EW calves were then separated from their dams and moved to a separate paddock on the same farm (Arcadia) on day 90 after birth, where they continued grazing the same pasture types and were manually (farm labourers) fed with a calf grower meal until calves were sold to the feedlot on day 205.

#### 2.1.4 Weaning application

Due to weaning the EW group at 90 days, fence-line weaning was initially implemented for 4 days on the Arcadia Farmland. Fence-line weaning is a low-stress technique where calves are separated from their mothers by a fence (Welk et al., 2024). This method allows cow-calf pairs to maintain visual and nose-to-nose contact, reducing calves' stress levels. Fence-line weaning is often considered more humane than abrupt weaning methods, as it allows calves to gradually transition away from relying on their dams for milk and social support; however, suckling is restricted (Whalin et al., 2021). Fence-line weaning was thus done to reduce weaning shock on dams and calves. To further enhance weaning adaptation, ten older calves (1-year-old) were placed among the group of EW calves, as the older calves had already adapted to feeding practices, helping the EW calves acclimate more effectively.

#### 2.1.5 Calf feeding

Moreover, calves in the EW group were subjected to a calf grower meal **(approximately 4kg per calf per day)** in addition to natural grazing pasture post-weaning (from 90 to 205 days of age) stocked at 2,5 ha/ LSU (Mokolobate, 2015). Calves were fed as a group of EW calves in their paddock by two permanent labourers responsible for rearing EW calves. Feed intake of the calf grower meal was not individually recorded, and thus, feed efficiency was not assessed. The calf grower meal was initially made available to EW calves at 3 weeks before weaning in a similar method to creep feeding, where they had access through creep gates while suckling their dams. After weaning, the ten older calves were placed mainly to familiarize EW calves with the creep feeding stations they were familiar with. The calf grower meal fed to EW calves, is a custom-mixed creep meal for small ruminants with 20 % protein that also supplies energy and minerals for the young animal’s development needs. It contains low levels of Non-Protein Nitrogen (NPN), as the young calf’s rumen is still developing and adapting to NPN feed, and the development of the enzyme is required for urea digestion (urease). It is palatable and contains sufficient grain to stimulate rumen development, as well as high levels of quality protein and a limited NPN content to increase weaning weight as fed with adequate roughage. Table 1 illustrates the dietary composition of the calf grower meal fed to EW calves.

**Table 1:** Early weaned calf grower meal nutrient composition (40 kg bag).

Nutrient	Unit (g/kg)
Protein	200
Urea (max)	20
Other NPN sources (max)	40
Total protein from NPN sources (max)	50.02 %
Moisture (max)	120
Calcium (min/max)	12/25
Phosphorus (min)	10
Energy MJME/kg (min) (estimated)	8.20
Vit A (IU/kg)	80 000

Source: Feed Composition Data (FCD) of calf grower meal fed to early weaned calves.

Notably, early exposure to creep feed enhanced feed intake after EW and calf grower meal was fed ad libitum to EW calves with unlimited feed access. Calf grower meal quantities were increased as calves grew older, requiring more feed for maintenance and bodily growth (Mare, 2018). Furthermore, Welk et al. (2024) stated that weaned calves require sufficient shade, and EW calves were provided with adequate shade to encourage optimal growth and development. Meanwhile, (CW) calves were allowed to suckle for 205 days and received no creep feeding or calf grower meal. CW calves were only subjected to their dam’s milk and natural grazing until weaning.

#### 2.1.6 Health management

Welk et al. (2024) also emphasized the importance of calf welfare immediately after weaning, indicating that beef cattle producers also need to cautiously manage their health programs to ensure optimal calf growth, ultimately determining the success of a weaning practice. Standard practice on Arcadia Farmland is where CW calves are treated for tapeworm twice before weaning at 205 days. In contrast, EW calves have a slight difference in health management. This differentiation entailed treating EW calves more regularly (approximately every 6 weeks) for internal parasites (tapeworms in particular) and boosters of electro guard (a vitamin, mineral, and amino acid supplement). This is done to treat and minimize stress-related symptoms, such as feed refusal, rumen stasis, dehydration, coccidiosis, diarrhea, adaptation constraints, and the development of rumen bacteria.

#### 2.1.7 Weighing and pregnancy diagnosis

**Fig. 1:**
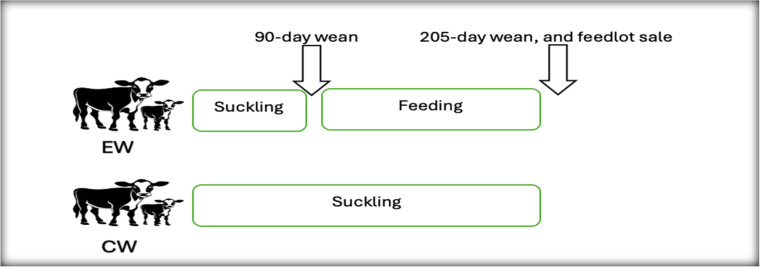
Diagram of weaning age application on the Arcadia farmland.

Source: Authors compilation.

All cow-calf pairs calves are weighed within 24 h postpartum to obtain the calf and dam weight at birth. Thereafter, EW calves are weighed twice, at 90 days of age and at a sale to the feedlot (day 205), while CW calves were only weighed at weaning and sold to the local feedlot at 205 days. Additionally, dams were exposed to bulls through natural mating approximately 60 days after calving. Pregnancy tests are conducted before weaning on day 205, and all non-pregnant dams are culled and sold to the feedlot and abattoir as slaughter heifers or feeder cattle (Spies, 2011). Therefore, the overall productivity per weaning practice was determined using the productive sub-model developed by Camargo et al. (2022). Fig. 1 illustrates the experimental management applied on the Arcadia Farmland from calving to 205-day sale to the local feedlot.

### 2.2 Methods

The productivity sub-model used in this study (Camargo et al., 2022) considered the total live weight of calves sold to the feedlot and the sale of non-pregnant dams after weaning per hectare of the total area (kg LW/ha). The following equation (Eq. (1)) was used:

(1) $$Productivity_{EW/CW}=\left(kg_{wm}+kg_{wf}+kg_{nd}\right)/A$$

Where: ***kg*** represents the Kilograms of live weight sold to the auction at 205 days; ***wm*** represents the male weaners sold while ***wf*** represents the female weaners sold; and ***nd*** the sale of non-pregnant dams after weaning and ***A*** = Total area in hectares where experimental herds were grazing and monitored.

Notably, Camargo et al. (2022) stated that calf weight at weaning or sale (205–210 days) is a pivotal contributor to the overall productivity of a cow-calf producer system. In addition, Whalin et al. (2021) explored key factors affecting the weaning age of calves and the subsequent productivity of cow-calf production systems. These factors included the birthweight of calves, the calving weight and age of dams, the weight ratio between dam and calf at birth, the dam’s inter-calving period (ICP), calf average daily gain (ADG), and weaning weight. Therefore, a regression analysis was done to explore how these productive factors significantly contributed to the weight of calves at 205 days after being subjected to early and conventional weaning practices.

#### 2.2.1 Regression analysis

The raw data was captured on a Microsoft Excel spreadsheet, and the relevant data was then coded for the analysis. Furthermore, the Statistical Package for Social Sciences (IBM SPSS Statistics 25) was used for the descriptive and regression analysis. Descriptive analysis was performed by observing the minimum, maximum, and mean variables on SPSS. Moreover, SPSS was also used to run one regression model to explore how cow-calf productive factors and variation in weaning practice (weaning early at 90 days vs. conventionally at 205 days) affect the live weight of calves sold to the feedlot at 205 days. Given the nature of the dependent variable, the General Linear Regression Model (GLM) was used to perform the analysis. The GLM was specified according to Gujarati (2004) using Eq. (2):

(2) $$Y_{i}=\beta_{0}+\beta_{1}X_{i}+\;\mu_{i}$$

Where$Y_{i}$, dependent variable (205-day live weight); $X_{i}$ vector of the productive factors; β, parameters to be estimated and$\;\mu_{i}$ as the Error term.

#### 2.2.2 Hypothesized explanatory variables

The explanatory variables hypothesized to influence calves' selling weight at 205 days are presented in Table 2, with each factor's expected direction of influence on the selling weight. The productive factors incorporated included Calf Birthweight, Dam Calving Weight, Cow-calf birth ratio, Dam Age at Calving in months, Dam Inter Calving Period (ICP), EW-Calf 90 Day Weight, EW-Calf 90 Day ADG, 205d ADG, and weaning strategy applied (early or conventionally) are expected to correlate with the weight of calves when sold to the feedlot at 205 days of age.

**Table 2:** Variables expected to influence 205-d weaning weight.

Productive Factor	Description	Expected sign for 205-d weight
Calf Birthweight (kg)	Measured weight of calve at birth	+
Weaning practice (90 vs 205 days)	The date at which calf suckling was entirely stopped	+
Dam Calving Weight (kg)	Measured weight of Dam after calving	+
Cow-calf birth ratio	The ratio of cow weight at calving to calve birthweight	+
Dam Age at Calving (months)	Age of dam (months) at calving	-/+
Dam first ICP (days)	The period between the first calf delivered and the subsequent calf	+
Calf 90-Day Weight (kg)	EW calf weight at 90 days	+
Calf 90d ADG (grams/day)	EW calf ADG after 90 days	+

**Note**: The variable weaning strategy was coded as a categorical dummy in the regression (0 = EW, 1 = CW).

Source: Compiled by the authors based on field data and variable definitions relevant to the study.

## 3 Results and discussion

The results are presented as follows:

1. Descriptive productive performance statistics and the Productivity sub-model results, comparing EW and CW practices.
2. The linear regression model results of productive factors affecting the 205-day weight of calves weaned early and conventionally

### 3.1 Descriptive productive performance statistics and productivity sub-model results, comparing EW and CW practices

Descriptive statistics were generated for each weaning group to reflect raw performance trends under farm conditions. These were not compared statistically but were included for the practical relevance of the study.

As seen in Table 3, CW-weaned calves weighed approximately 33.58 kg at birth, while their dam weight at birth averaged 481.19 kg. When expressed as a ratio, the cow-calf birth ratio of CW calves averaged 7:1, indicating that calves weighed 7 % of their dam’s weight at birth.

**Table 3:** Productive performance statistics of cow-calf pairs subjected to CW practices.

	N	Min	Max	Mean		Std. Deviation
				Statistic	Std. Error	
Calf Birthweight (kg)	76	27	42	**33.68**	.38	3.42
Dam Calving Weight (kg)	76	350	600	**481.19**	5.85	52.04
Cow-calf birth ratio	76	5	10	**7.09**	.12	1.03
Dam Age at Calving (months)	76	28	38	**32.09**	.56	5.01
Dam ICP (days)	76	375	660	**419.25**	6.36	56.52
205d WW (kg)	76	209	266	**237.11**	1.57	14.01
205d ADG (grams/day)	76	854	1098	**992.32**	7.32	65.07

**Note**: CW refers to conventional weaning (205 days).

Source: Authors’ calculations based on data collected from Arcadia Farmland.

According to Johanson and Berger (2003), the cow-calf birth ratio is a fundamental predictor of calving ease and perinatal mortalities. A >10 % ratio is deemed an enabling factor to dystocia and stillbirth in cow-calf systems (Johanson & Berger, 2003). Thus, this factor is an essential component that producers must monitor to ensure long-term productivity, which refers to managing calf survival from birth to weaning and sale. Dam age at calving (months) and their ICP (days) averaged 32 and 419 days, respectively (Table 3). Arthington and Minton (2004) emphasized dam age at calving as a key component of calf-rearing ability, as producers should allow dams to calve at mature ages for optimal calf rearing, which may lead to heavier calves at weaning. Moreover, when looking at the ADG and weaning weight, CW calves weighed 237 kg with an average daily gain of 992 g/d at 205 days when sold to the feedlot. The higher 205-day weaning weight in CW is extremely important, especially for heifer calves (Taylor et al., 2021). When producers retain weaned heifer calves for replacement, extended suckling (CW) may promote sufficient early growth to support future reproductive performance. Table 4 illustrates the correlation between the various productive factors in the Conventional Weaning (CW) group.

**Table 4:** Correlation between productive factors (conventionally weaned group).

Productive factor	Calf birth weight	Dam weight at birth	CC-birth ratio	Dam age at calving	Dam ICP	Calf 205-day weight	Calf 205-day ADG
**Calf birth weight (kg)**	**1**						
**Dam weight at birth (kg)**	0.06	**1**					
**CC-birth ratio**	**0.63**	**−0.72**	**1**				
**Dam age at calving (months)**	0.07	0.27	−0.16	**1**			
**Dam ICP (days)**	0.06	0.31	−0.2	−0.08	**1**		
**Calf 205-day weight (kg)**	0.32	0.22	0.06	0.00	0.11	**1**	
**Calf 205-day ADG (grams/day)**	0.08	0.22	−0.1	−0.02	0.1	**0.97**	**1**

**Note**: Cells highlighted in green represent strong (close proximity to +1) positive correlation coefficients, while cells highlighted in red represent strong negative correlations (close proximity to −1).

Source: Authors’ calculations based on data collected from Arcadia Farmland.

Table 4 also reveals a strong positive correlation **(closer to +1)** between calf 205-day weight and Calf 205-day ADG (0.97). This is expected since Average Daily Gain (ADG) directly influences 205-day weight (Story et al., 2000).

A strong positive correlation (0.63) between cow-calf birth ratio and calf birth weight indicates that heavier calves increase the ratio. Conversely, the strong negative **(closer to −1)** correlation (−0.72) between dam weight at calving and cow-calf birth ratio reflects that heavier dams lower the ratio, as it is calculated with dam weight as the denominator. Thus, the ratio increases when calf birth weight rises while dam weight remains constant and decreases when dam weight increases. These findings have important implications for South African cattle producers. A higher cow-calf birth ratio suggests increased physiological demand on the dam at birth, which may impact calving ease, particularly in first-time calving heifers. This observation corroborates findings by Johanson and Berger (2003). For producers selecting optimal birth weights, balancing calf growth potential with dam size is essential, especially under extensive grazing, where maintaining optimal bodyweight is crucial for reproduction and herd efficiency. The results highlight the importance of dam bodyweight management at calving, as it directly influences calf growth and weaning outcomes. From a herd management perspective, maintaining an optimal dam weight at calving could enhance weaning weights and production efficiency. However, while dam weight appears to predict calf performance strongly, other factors such as **genetics**, nutrition, and environmental conditions should also be considered when optimizing weaning strategies. Overall, the majority (76 %) of the productive factors within the CW group have a positive correlation. Table 5 provides the productive performance statistics of EW cow-calf pairs

**Table 5:** Productive performance statistics of cow-calf pairs subjected to EW practices.

Productive factor	N	Min	Max	Mean		Std. Deviation
				Statistic	Std. Error	
Calf Birthweight (kg)	76	18	37	**28.04**	.44	3.90
Dam Calving Weight (kg)	76	260	480	**380.20**	4.98	43.49
Cow-calf birth ratio	76	4	16	**7.52**	.19	1.67
Dam Age at Calving (months)	76	24	35	**27.62**	.26	2.33
Dam ICP (days)	76	340	461	**347.59**	11.28	98.37
Calf 90-Day Weight (kg)	76	86	118	**102.92**	.85	7.40
90d ADG (grams/day)	76	540	880	**748.82**	7.45	64.97
205d WW (kg)	76	137	261	**210.38**	2.15	18.75
205d ADG (grams/day)	76	527	1141	**889.45**	10.20	89.00

**Note**: EW refers to early weaning (90 days).

Source: Authors’ calculations based on data collected from Arcadia Farmland.

According to Table 5, EW-weaned calves weighed approximately 28 kg at birth, while their dam weight at birth averaged 380.20 kg. Based on the farm productive data, EW calves weighed 5 kg less at birth than CW calves. The lower weight at birth may be due to EW calf dams having a lower weight and age. This observation confirms findings by Johanson and Berger (2003) and Šlyžiene et al.(2023), who found that dams with a lower weight and age at birth produce a lighter calf at birth, possibly influencing the calf's long-term productivity. Regarding the cow-calf birth ratio, EW calves had a low ratio of 4.23:1 (Johanson & Berger, 2003). A low cow-calf birth ratio is an advantage relating to calving ease and decreased perinatal mortalities, especially with the EW group having younger and lighter dams upon calving (Johanson & Berger, 2003). The cow-calf birth ratio is a fundamental factor that producers must consider for productivity. This indicates that under South African conditions, beef cattle producers may decide to wean calves early when dams are younger and lighter in weight to ensure young dams' long-term productivity, validating the results by Meagher et al. (2019) and Camargo et al. (2022).

The ICP (days) of dams subjected to EW averaged approximately 347 days. This indicates that weaning calves shortens dams' ICP, allowing earlier exposure of dams to the bull. A short ICP, when appropriately managed, can be a significant advantage of EW (Story et al., 2000; Meagher et al., 2019 and Taylor et al., 2021), as it supports optimal herd productivity and economic viability. According to Rust and Rust (2013), a short ICP in cattle boosts herd productivity by increasing calf output and enhancing economic returns. It improves overall herd efficiency and allows for rapid genetic advancements while ensuring better resource utilization. This shows that regular calving helps maintain consistent cow condition, reduces maintenance costs, and supports steady profitability, confirming the results of Taylor et al. (2021).

Moreover, EW calves weighed 210 kg on average at 205 days of age, with an ADG of 889.45 g/d at 205 days when sold to the feedlot. These observations resemble those of Julian & Tess (2002), Guimaraes et al. (2006), and Camargo et al. (2022). However, Savage (2011) and Tatham et al. (2004) argued that EW can increase calf weaning weights when the weaning season under East Tennessee (United States of America) circumstances is adjusted to suit adequate rainfall for increased forage availability for grazing. This emphasizes the geographical and climatic differences among countries (Vaz et al., 2022), highlighting the contribution of this study under local conditions for South African producers. Moreover, the authors (Hudson et al., 2010) mentioned that a greater ADG among EW calves stems from an improved FCR. This is due to EW calves being able to convert feed more efficiently to live weight, ultimately increasing the productivity of EW within cow-calf systems.

As seen in Table 6, there is a very strong positive correlation (0.978) between calf 205-day weight and calf 205-day ADG, confirming that higher growth rates lead to heavier weaning weights in the EW group, which is in line with the results of Arthington and Minton (2004). Similarly, the strong correlation (0.85) between calf 90-day weight and calf 90-day ADG indicates that early weight advantages contribute to improved growth performance. The positive correlation (0.76) between calf birth weight and cow-calf birth ratio suggests that heavier calves increase the ratio, as birth weight is the numerator. In contrast, the strong negative correlation (−0.75) between dam weight at birth and cow-calf birth ratio reflects that heavier dams result in lower ratios due to the denominator effect. This highlights the influence of maternal size on birth weight proportions, reinforcing the importance of balancing calf growth potential with dam bodyweight in early weaning systems. Notably, dam weight at birth and cow-calf-birth ratio (−0.75 in early vs. −0.72 in conventional) are very similar in both systems, indicating a consistent trade-off between dam weight and cow-calf birth ratio as highlighted by Johansen and Berger (2003). Overall, the majority (75 %) of the factors have a positive correlation.

**Table 6:** Correlation of productive factors (early weaned group).

	Calf birth weight	Dam weight at birth	CC-birth ratio	Dam age at calving	Dam ICP	Calf 90-day weight	Calf 90-day ADG	Calf 205-day weight	Calf 205-day ADG
Calf birth weight (kg)	**1**								
Dam weight at birth (kg)	−0.20	**1**							
CC-birth ratio	**0.76**	**−0.75**	**1**						
Dam age at calving (months)	0.03	0.28	−0.16	**1**					
Dam ICP (days)	0.01	0.15	−0.07	−0.03	**1**				
Calf 90-day weight (kg)	0.48	−0.13	0.40	0.13	0.11	**1**			
Calf 90-day ADG (grams/day)	−0.05	−0.03	0.00	0.13	0.12	**0.85**	**1**		
Calf 205-day weight (kg)	0.23	0.19	0.01	0.26	0.04	0.41	0.32	**1**	
Calf 205-day ADG (grams/day)	0.02	0.24	−0.16	0.26	0.04	0.31	0.34	**0.98**	1

**Note**: Cells highlighted in green represent strong (close proximity to +1) positive correlation coefficients, while cells highlighted in red represent strong negative correlations (close proximity to −1).

Source: Authors’ calculations based on data collected from Arcadia Farmland.

Furthermore, when observing the individual calf data set, it was seen that there is a more uniform ADG-to-weaning weight relationship for CW calves, while a more heterogeneous or non-uniformed relationship is observed for EW calves. The more uniform ADG-to-weaning weight relationship observed in CW calves likely reflects their prolonged access to maternal milk and gradual adaptation to solid feed, resulting in more consistent growth patterns. In contrast, EW calves experienced an abrupt transition to a solid-feed-based diet at a younger age, making them more susceptible to variations in nutritional intake, stress responses, and adaptation rates, leading to a more heterogeneous growth pattern. Environmental and management factors, such as feed quality and availability, may further amplify these differences (Savage, 2011).

A productivity sub-model determined the total amount of live weight produced per ha between two weaning practices on day 205. The productivity sub-model used (Camargo et al., 2022) considered the total live weight of calves sold to the feedlot (calf weight on day 205) and the sale of non-pregnant dams after weaning per hectare of the total area (kg LW/ha). Table 7 presents descriptive productivity model outputs based on farm data, which are not intended for inferential statistical comparison.

**Table 7:** Productivity sub-model results.

	Weaning practice	
	Early Weaning (EW)	Conventional Weaning (CW)
Number of male weaners sold	37	38
Number of female weaners sold	39	38
Number of non-pregnant heifers	5 (6 %)	12 (16 %)
Kilograms of live weight sold weaner males (kg)	8103	9120
Kilograms of live weight sold weaner females (kg)	7839	8854
Kilograms of live weight sold from non-pregnant dams (kg)	2150	5160
Total live weight on day 205(kg)	**18,092**	**23,134**
Total productivity per ha (kg weaners /ha)	**91**	**115**
Total live weight margin when sold to the feedlot on day 205 (kg)	**CW >EW by 5042**	
Total productivity margin per ha (kg)	**CW >EW by 24**	

Source: Authors’ calculations based on farm data from the Farmland.

The results from the productivity sub-model (Table 7) demonstrate that CW (weaning calves at 205 days) resulted in 5042 kg more total live weight and 24 kg more live weight per hectare compared to EW (weaning at 90 days). A **major contributor** to this difference was the higher live weight sold from **non-pregnant dams** under CW (5160 kg) compared to EW (2150 kg). This indicates that the culling of 12 non-pregnant CW dams, versus 5 in the EW group, increased the total live weight marketed by the CW group. Additionally, parity differences between the groups must be considered. CW included several second-parity cows, whereas EW exclusively consisted of primiparous heifers. Second-parity dams typically show greater maternal performance, including higher milk production and better calf-rearing outcomes (Taylor et al., 2021). This inherent physiological advantage likely contributed to the higher live weight production observed under CW. Therefore, the greater productivity observed under CW is not solely attributable to calf growth but also reflects **increased cull cow contribution** and **maturity-related advantages** in dam performance.

Conversely, including first-time heifers in the EW group may have limited calf weight gain, as primiparous cows are still allocating significant energy to their growth and recovery postpartum (Tatham et al., 2004). Despite these challenges, the EW group achieved 94 % pregnancy rates compared to 84 % in the CW group, showcasing the benefits of reduced lactation demands on reproductive performance. By weaning calves earlier, EW reduces the energy-intensive process of lactation, enabling heifers to recover bodyweight more quickly and improving fertility. This aligns with the findings of Camargo et al. (2022), who reported that EW enhances dam fertility by minimizing the energy drain associated with lactation. In South African dry regions, where nutritional stress is prevalent, the ability of EW to improve conception rates offers a strategic advantage for producers aiming to optimize reproductive performance.

The absence of a suckling calf in EW systems also removes hormonal inhibitors of ovulation, further boosting reproductive efficiency (Taylor et al., 2021). These findings suggest that while CW maximizes live weight production per hectare, EW enhances reproductive performance, particularly where forage availability and quality are limiting. For producers considering EW, particularly in primiparous heifers, these results highlight its potential to improve herd reproductive efficiency in drought-prone environments characteristic of South Africa.

### 3.2 Linear regression results

The General Linear Model (GLM) regression was employed to investigate the influence of selected productive factors on the 205-day weight of calves under early (EW) and conventional (CW) weaning practices. The model included key cow-calf performance indicators: calf birthweight, dam calving weight, dam age at calving, cow-calf birth ratio, dam ICP, calf 90-day weight, calf 90-day ADG, and the weaning practice cow-calf pairs were subjected to. The variable **weaning practice** was coded as a categorical dummy (0= EW, 1= CW). Notably, the model summary showed that the regression model was overall significant, with a p-value of 0.00942. This indicates that the group of productive factors reliably predicted the weight of calves at 205 days of age (Gujarati, 2004). As seen in Table 8, the adjusted R-squared showed that the independent variables represent 55 percent of the variation in 205-day weight of calves. This suggests a statistical relationship between the variables and indicates that the model fits the data well (Gujarati, 2004).

**Table 8:** GLM regression model summary.

Model	R-Square	Adjusted R-Square	Std. Error of the Estimate	F-Change	df_1_	Sig. F	Durbin-Watson
GLM	0.579	0.548	.049	114,597.7	141	*0.00942	1.088

Source: GLM regression.

#### 3.2.1 GLM regression results for cow-calf productive factors associated with the 205-day weight of calves

The regression analysis of productive factors revealed that Dam Calving Weight (*p* = 0.0034) and the weaning practice applied, early or conventional (*p* = 0.0415), were statistically significant at a 1 % and 5 % significance level. Other variables, including calf birthweight and 90-day performance indicators, were not significant predictors of 205-day weight in the model, suggesting their effects may be more localized within specific weaning strategies rather than across the entire dataset.

### 3.3 Interpretation of significant predictors

• ***Dam Calving Weight***

The model revealed a statistically significant positive relationship between dam calving weight and 205-day calf weight (Table 9). This finding is supported by previous literature (Johanson & Berger, 2003; Šlyžienė et al., 2023), indicating that heavier dams tend to have higher body reserves, improved lactation performance, and stronger maternal capacity. These characteristics directly enhance calf growth, especially during early postnatal development. From a biological perspective, heavier cows typically have greater fat and muscle reserves at calving, enabling them to allocate more energy to milk production during lactation. This enhanced milk yield and quality supports calf weight gain, particularly in the first 90 to 120 days post-calving when milk remains the primary nutrient source, as found by Whalin et al. (2021). It is important to note that because the EW group predominantly consisted of younger, primiparous heifers, their calves were more reliant on lower milk production and supplementary feeding during the critical early postnatal period. This may have constrained the growth potential of calves in the EW group, making the influence of dam age more pronounced (Taylor et al., 2021). Ultimately, the observed significance across both EW and CW calves underscores dam calving weight as a universal driver of weaning weight, irrespective of the weaning management strategy.

**Table 9:** GLM regression results for cow-calf productive factors associated with the 205-day weight of calves.

	Unstandardized Coefficients			
Productive Factor	B	Std Error	t-stat	*p*-value
**Constant**	205.97	37.29	5.52	.0012
**Calf Birthweight (kg)**	1.348	.883	1.527	.129
**Dam Calving Weight (kg)**	0.025	0.67	−0.375	0.0034*
**Cow-calf birth ratio**	−3.083	3.310	−0.931	.353
**Dam Age Calving (months)**	−0.071	.249	.215	.830
**Dam ICP (days)**	0.001	.015	.41	.681
**90d WW (kg)**	0.251	.776	.54	.774
**90d ADG (kg)**	0.061	0.079	.777	.483
**WeanPrac(1=CW;0=EW)**	−82.195	33.149	−2.459	0.0415**

**Note:** * and ** represent statistical significance at 1 % and 5 % respectively.

CW refers to conventional weaning (205 days); EW refers to early weaning (90 days).

Source: GLM regression results.

Furthermore, while cow-calf birth ratio was not statistically significant in the model, it is essential to recognize that dam weight also indirectly affects this ratio. In previous studies, higher dam calving weight was associated with more favorable cow-calf birth ratios, which were, in turn, positively linked to calf growth potential (Vaz et al., 2022).

*Practical Implications for Producers’ Weaning Age Decisions:*

These findings reinforce the importance of pre-calving dam nutrition and weight management for South African beef producers. Maintaining optimal calving weights, particularly in first- and second-parity cows, is essential to ensuring strong calf performance, especially under early weaning systems where calves depend more on early milk yield before transitioning to creep and solid feeds.

• ***Weaning Practice (EW* vs. *CW)***

The model also confirmed the **weaning strategy as a significant predictor (5 %)** of 205-day weight, with the farm data indicating that CW calves weighed approximately 27 kg more on average than EW calves at the same age. This finding is consistent with several prior studies (Taylor et al., 202 and Hudson et al., 2010), which consistently show that calves allowed suckling for more extended periods (e.g., 205 days) demonstrate superior weaning weights due to uninterrupted access to maternal milk and lower early-life nutritional stress. In contrast, EW calves are removed from their dams at 90 days, a period when rumen function is still developing and reliance on solid feed is only beginning. While EW calves in this study were supplemented with calf grower meal and had early exposure to creep feeding, the performance gap suggests that extended suckling under CW still provides a competitive growth advantage, at least in terms of weight at the point of sale to the feedlot.

This does not negate the strategic value of EW, especially in herds where reproductive efficiency and dam energy balance are the priority. However, the trade-off in reduced weaning weight must be carefully managed through optimized creep feeding programs and careful attention to early calf health, rumen development, and forage quality post-weaning.

*Practical Implications for South African Beef Cattle Producers’ Weaning Age Decisions:*

For producers evaluating whether to adopt EW or CW strategies, the results suggest that while CW may deliver higher market weights at 205 days, EW may still offer benefits in terms of dam reproductive recovery and overall herd efficiency, particularly when applied to primiparous heifers (Rust and Rust, 2014). In environments like South Africa, where feed availability fluctuates and drought cycles impact dam condition, EW may support better long-term herd fertility. However, effective calf nutrition planning will be required to minimize weight losses relative to CW systems. These results provide strong guidance to South African beef producers on optimizing both **dam calving weight** and **weaning management** to enhance system productivity while also highlighting the importance of balancing short-term growth outcomes with long-term reproductive goals*.*

## 4 Conclusions

This study investigated the key factors influencing the 205-day weight of calves subjected to early (EW) and conventional (CW) weaning practices under South African beef production systems. The results revealed distinct differences in productive factors between the two weaning systems, highlighting the importance of context-specific management practices. The analysis showed that only **weaning practice** and **dam calving weight** among the evaluated variables were statistically significant predictors of calf 205-day weight.

The farm performance data demonstrated that calves weaned conventionally at 205 days (CW) were heavier than those weaned early at 90 days (EW) by an average of 27 kg per calf. This confirms the substantial impact that extended access to maternal milk, and suckling has on calf growth and final sale weight. However, while CW systems improve immediate weight outcomes, they must be balanced against other operational priorities, including dam reproductive recovery, forage availability, and herd fertility management. Although associated with lower weaning weights, EW systems may offer strategic advantages in managing energy balance in primiparous heifers and supporting long-term reproductive goals. EW practices shortened the inter-calving period (ICP) and improved dam fertility, ultimately enhancing herd reproductive efficiency, making it a potentially viable strategy under unfavorable climatic and drought-prone regions characteristic to South Africa. These findings highlighted the trade-offs between weaning systems, with CW favouring higher total live weight production and EW supporting long-term reproductive efficiency.

Additionally, the positive association between **dam calving weight** and calf weaning weight underscores the critical role of maternal body condition in supporting calf performance. Heavier dams at calving likely produce more milk and provide better early maternal support, both of which are essential to calf growth, especially under early weaning conditions where calves transition to solid feed earlier. While other factors such as calf birthweight, dam age, and early calf performance (e.g., 90-day ADG) showed expected trends, they did not emerge as statistically significant in the regression model, likely due to the overriding influence of weaning strategy and dam body condition. This study represented one of the first attempts to evaluate and report early weaning (EW) practices at 90 days in South African beef cattle.

## 5 Recommendations for south african beef cattle producers

Based on the study findings, we recommend the following:

### 5.1 When to adopt early weaning (EW)

1. Drought or Resource Constraints: EW is ideal when facing drought or limited forage, which is common in the South African region. This is due to it reducing the dam’s nutritional load and supporting carrying capacity at the time.
2. Improving Reproductive Efficiency: EW can enhance herd fertility by reducing ICP, making it a good strategy for boosting long-term reproductive performance.
3. Financial Liquidity: EW may allow for earlier sales of calves, providing quicker financial returns, even if calves are lighter.
4. Managing Early Bred First-Time Calving Heifers: EW is beneficial for first-time calving (primiparous) heifers, as it helps manage their energy demands and enables better overall health, allowing them to be bred early (14–15 months) without compromising their immediate development or future productivity.

### 5.2 When to continue conventional weaning (CW)

1. Maximizing Productivity: CW produced heavier calves, maximizing live weight per hectare, making it the better choice for farms or areas with consistent forage quality and availability.
2. Replacement Heifer Development: When developing replacement heifers to become efficient, productive dams, CW allows calves to gain maximum weight before weaning compared to EW. This ensures that they are in optimal condition to conceive, particularly when early mating (14–15 months) is implemented to maximize the dam's long-term production.
3. Optimal Calf Growth: CW allows calves to benefit from extended lactation, leading to better overall growth and market (live weight) value at approximately 205-day sale for improved farm financial performance.

### 5.3 Broader considerations and being flexible

1. **Prioritize Pre-Calving Dam Nutrition**: Maintaining optimal dam body weight at calving is essential for calf growth, regardless of the weaning strategy. Producers should focus on pre-calving supplementation, especially for younger or first-parity cows.
2. **Select Weaning Strategy Based on Production Goals**: CW improves weaning weight but may increase dam energy demand and delay rebreeding. EW may benefit herd fertility and dam recovery, particularly in resource-limited or drought-prone systems. However, targeted post-weaning nutrition programs must be in place to support EW calf growth.
3. **Balance Herd Structure and Feeding Programs**: When applying EW, producers should ensure adequate feeding infrastructure, such as creep feeding and grower rations, to minimize weight disparities at sale. Managing dam age and parity structure within the herd will also influence long-term productivity.

## 6 Limitations of this study

A noteworthy limitation of the experimental design is the inclusion of second-time calvers in the Conventional Weaning (W205) group, while the Early Weaning (W90) group comprised exclusively primiparous cows. This disparity may influence comparisons, as second-time calvers have different physiological demands and productive potentials than first-time heifers. Moreover, the sample was farm-specific and not randomly drawn from the broader beef cattle population in South Africa. Results may vary across regions or management practices in South Africa, and producers are encouraged to adapt these strategies to their specific environmental, financial, and operational conditions. Although this study was conducted on a closed stud herd (Arcadia Bonsmara), detailed pedigree information or breeding values were unavailable for analysis. As such, the study did not account for specific genetic or non-genetic factors beyond those captured in the raw farm performance data. While this reflects the decision-making reality of many commercial producers, future research could enhance accuracy by integrating pedigree records and genetic evaluations to isolate heritable influences on productive performance.

Nevertheless, while this study introduces variability, the primary objective was to establish baseline data for EW practices in South Africa. By presenting this data, we aim to stimulate further research and provide preliminary insights on weaning age decision-making for South African farmers considering EW. Future studies should strive to comprehensively evaluate EW and CW strategies within groups of the same parity to minimize confounding effects. Such analysis should also include a multi-year data collection to refine weaning decisions further. Additionally, future studies should assess these weaning practices using varying breeds and management and feeding strategies across South Africa.

## CRediT authorship contribution statement

**Brent Damian Jammer:** Visualization, Validation, Supervision, Software, Resources, Project administration, Methodology, Investigation, Funding acquisition, Formal analysis, Data curation, Conceptualization. **Willem Abraham Lombard:** Writing – review & editing, Writing – original draft, Visualization, Validation, Supervision, Software, Resources, Project administration, Methodology, Investigation, Funding acquisition, Formal analysis, Data curation, Conceptualization. **Henry Jordaan:** Writing – review & editing, Writing – original draft, Visualization, Validation, Supervision, Software, Resources, Project administration, Methodology, Investigation, Funding acquisition, Formal analysis, Data curation, Conceptualization.

## Declaration of competing interest

The authors declare that they have no known competing financial interests or personal relationships that could have appeared to influence the work reported in this paper.
